# Different sampling strategies for optimal detection of the overall genetic diversity of methicillin-resistant *Staphylococcus aureus*

**DOI:** 10.1128/spectrum.00140-24

**Published:** 2024-05-29

**Authors:** Tjasa Zohar Cretnik, Leon Maric, Maja Rupnik, Sandra Janezic

**Affiliations:** 1 National Laboratory of Health, Environment and Food, Maribor, Slovenia; 2 Faculty of Medicine, University of Maribor, Maribor, Slovenia; University of Calgary, Calgary, Alberta, Canada

**Keywords:** MRSA, clonal structure, blood stream infections, surveillance, resistance, virulence

## Abstract

Surveillance schemes for methicillin-resistant *Staphylococcus aureus* (MRSA) are widely established at the national and international levels. Due to the simple standardization of the protocol, mainly isolates from bloodstream infections are used. However, the limitations of this simple surveillance system are well described. We conducted a comprehensive analysis of MRSA isolates in a large Slovenian region over 5 years to identify the optimal sample group for assessing the overall MRSA diversity. At the same time, this study provides to date non-available molecular characterization of Slovenian MRSA isolates. A total of 306 MRSA isolates from various sources were sequenced and phenotypically tested for resistance. The isolates exhibited significant molecular diversity, encompassing 30 multi locus sequence type (MLST) sequence types (STs), 39 ST-SCC*mec* genetic lineages, 49 *spa* types, and 29 antibiotic resistance profiles. Furthermore, the isolate pool comprised 57 resistance genes, representing 22 resistance mechanisms, and 96 virulence genes. While bloodstream isolates, commonly used in surveillance, provided insights into frequently detected clones, they overlooked majority of clones and important virulence and resistance genes. Blood culture isolates detected 21.3% *spa* types, 24.1% resistance phenotypes, and 28.2% MLST-SCC*mec* profiles. In contrast, strains from soft tissues demonstrated superior genomic diversity capture, with 65.3% *spa* types, 58.6% resistance phenotypes, and 71.8% MLST-SCC*mec* profiles. These strains also encompassed 100.0% of virulence and 82.5% of resistance genes, making them better candidates for inclusion in surveillance programs. This study highlights the limitations of relying solely on bloodstream isolates in MRSA surveillance and suggests incorporating strains from soft tissues to obtain a more comprehensive understanding of the epidemiology of MRSA.

**IMPORTANCE:**

In this study, we investigated the diversity of methicillin-resistant *Staphylococcus aureus* (MRSA), a bacterium that can cause infections that are difficult to treat due to its resistance to antimicrobial agents. Currently, surveillance programs for MRSA mainly rely on isolates from bloodstream infections, employing a standardized protocol. However, this study highlights the limitations of this approach and introduces a more comprehensive method. The main goal was to determine which group of samples is best suited to understand the overall diversity of MRSA and to provide, for the first time, molecular characterization of Slovenian MRSA isolates. Our results suggest that including MRSA strains from soft tissue infections rather than just blood infections provides a more accurate and comprehensive view of bacterial diversity and characteristics. This insight is valuable for improving the effectiveness of surveillance programs and for developing strategies to better manage MRSA infections.

## INTRODUCTION

Methicillin-resistant *Staphylococcus aureus* (MRSA) is recognized as an important health threat to patients and healthcare systems by public health authorities all over the world (1–3). Despite growing efforts to reduce the spread of MRSA, its burden continues to increase. It causes more than 100,000 deaths attributable to antimicrobial resistance annually globally (4, 5). In accordance with the latest report from SENTRY, the proportions of MRSA among all *S. aureus* isolates and among soft tissue infections isolates in 33 countries worldwide range from 5.0 to 61.1% and 5.5 to 66.7%, respectively (https://sentry-mvp.jmilabs.com/, retrieved 1 April 2024). The proportions of MRSA among blood culture isolates ranged from 0% to 82% (6), from 0% to 64.3% (https://sentry-mvp.jmilabs.com/, retrieved 1 April 2024), and from 1.1% to 50.8% (3).

A robust and periodically evaluated surveillance system is needed to correctly assess the burden of infections caused by MRSA and to get insight into the epidemiology and genetic variability (7–11). Furthermore, effective diagnostics and laboratory capacity for pathogen and genomic surveillance is one of three pillars of integrated surveillance identified by the World Health Organization as a core concept for strengthening health emergency preparedness, response, and resilience (12). Incorporation of whole-genome sequencing (WGS) into national and international monitoring programs is also recommended because of its ability to fully characterize emerging high-risk clones and otherwise greatly enhance the quality and quantity of data on the genetic traits of microorganisms (13).

Most local, national, and international MRSA surveillance systems rely on data derived from analyzing and studying only blood culture isolates (5, 6, 14). As they represent the top of the iceberg of MRSA infections that are limited to the hospital environment, insight into the clonal structure, virulence, and resistance profiles and estimates of burden of infections are consequently biased (15, 16). Some countries have already decided to add other groups of samples to their surveillance systems, but it is not clear to what extent (14). Generally, it is not well understood which group of isolates or their combinations are the most informative to better understand MRSA epidemiology. Furthermore, without a unified sampling system, the comparison of data and identification of the drivers of success of different clones are not possible (17).

In Slovenia, the proportions of MRSA isolates among *S. aureus* isolates from blood cultures and from all clinical isolates have been rather stable for the last two decades, varying between 6% and 10% (18, 19). The highest proportion of invasive MRSA isolates (21.3%) was reported in 2000 and the lowest (6%) in 2012 and 2013. A systematic national molecular surveillance of MRSA has not yet been established.

The aim of this study was to acquire data on the clonal structure of MRSA populations on a well-defined collection of isolates from a 5-year observational period and to create a collection of sequences for continuous prospective molecular surveillance of MRSA at a national level, based on whole-genome sequencing. By comparing the informative values of different groups of isolates, we aimed to identify candidate sample group(s) that would best cover the overall genomic diversity. This could also enhance monitoring programs at an international level.

## RESULTS

In total, 306 MRSA strains were examined, comprising samples from surveillance and four distinct clinically relevant groups. The analysis involved assessing multi locus sequence type (MLST) sequence types (STs), *spa* types, antibiotic resistance, resistome, and virulence profiles. Following the determination of the overall genomic diversity within the strain collection, we conducted comparisons among different sample groups to understand the coverage of strain diversity by each individual group or their combinations.

### SCC*mec* type and *mec* gene distribution

The presence of the *mec*A and *mec*C genes was confirmed in 301 (98.0%) and 5 (1.6%) isolates, respectively. Among 301 isolates with *mecA* gene, 10 types of staphylococcal cassette chromosome *mec* (SCC*mec*) were detected (Table 1). The most prevalent SCC*mec* type was IIa detected in 186 (60.8%) isolates, followed by type IVa in 64 (20.9%) and type IVc in 23 (7.5%) isolates. Other types were present in 0.3% (type I) to 2.9% (type Vc) isolates. In two isolates, SCC*mec* type could not be determined. All 10 types of SCC*mec* were present among the non-blood clinical isolates and six types among the blood culture isolates (Table 1).

**TABLE 1:** Distribution of SCC*mec* types among different groups of isolates^*a*^

SCC*mec*	% BCI (*n* = 36)	% SCI (*n* = 137)	NBCI (*n* = 133)				*mecA* and *mecC* positive (*n* = 306)	
			% all NBCI	% STI (*n* = 78)	% RTI (*n* = 35)	% UTI (*n* = 20)	%	*n*
Ia	0.0	0.0	0.8	1.3	0.0	0.0	0.3	1
IIa	66.7	67.9	51.9	43.6	68.6	55.0	60.8	186
IV	0.0	0.0	1.5	2.6	0.0	0.0	0.7	2
IVa	19.4	20.4	21.8	20.5	17.1	35.0	20.9	64
IVb	0.0	0.7	0.8	1.3	0.0	0.0	0.7	2
IVc	5.6	5.1	10.5	16.7	2.9	0.0	7.5	23
IVh	2.8	2.2	2.3	0.0	2.9	10.0	2.3	7
V	0.0	0.0	3.8	5.1	2.9	0.0	1.6	5
Vc	2.8	2.2	3.8	3.8	5.7	0.0	2.9	9
XI	2.8	0.0	3.0	5.1	0.0	0.0	1.6	5
Not detected	0.0	1.5	0.0	0.0	0.0	0.0	0.7	2

^*a*^ SCC*mec*, *staphylococcal* cassette chromosome *mec*; BCI, blood culture isolates; SCI, isolates from surveillance cultures; STI, isolates from soft tissues; RTI, isolates from respiratory tract; UTI, isolates from urinary tract; NBCI, non-blood clinical isolates.

### Clonal diversity and distribution

Of the 30 different MLST STs, 198 (64.7%) isolates belonged to three representatives of CC5, 104 (34.0%) to ST5, 82 (26.8%) to ST225, and 12 (3.9%) to ST2883 (CC5). Twenty-six (8.5%) isolates belonged to ST22 (CC22), 17 (5.6%) to ST1 (CC1), and 14 (4.6%) to ST97 (CC97). The other 24 sequence types were represented by less or equal to 2% of all the isolates (Table S1). Nine MLST STs, ST7821, ST7822, ST7824, ST7826-7828 (CC5), ST7825 (CC1), ST7823 (CC8), and ST7829, were described for the first time. A single isolate with the ST7829 profile was a *mecC-*positive singleton.

Three hundred and four isolates were assigned to 39 ST-SSC*mec* genetic lineages (Table 2). Strains carrying SCC*mec* types I and II were regarded as hospital-acquired MRSA (HA-MRSA) strains. Strains carrying SCC*mec* types IV, V, and XI were regarded as community-acquired MRSA (CA-MRSA) or livestock-associated MRSA (LA-MRSA) in accordance with the accepted clone characterization (20). The Ia and IIa SCC*mec* types (HA-MRSA clones) were present in 187 isolates (61.1%) pertaining to 10 genetic lineages. ST5-IIa and its single locus variant at the *tpi* locus ST225-IIa, both belonging to the pandemic CC5 clonal complex, were represented by 85 (27.8%) and 81 (26.5%) isolates, respectively. One isolate was typed as ST228-I known also as a South German clone. Isolates with ST1-IVa, ST97-IVa, ST130-XI, ST398-Vc, and ST5-IVc profiles were regarded as LA-MRSA and included 54 isolates (17.7%). Of the 63 CA-MRSA isolates (20.6%) with 23 different ST-SCC*mec* profiles, 51 belonged to six well-established European CA-MRSA clones (ST5-IV, ST8-IV, ST22-IV, ST30-IV, ST88-IV, and ST45-IV).

**TABLE 2:** Distribution of MLST-SCC*mec* types, pertaining clonal complexes and *spa* types among different groups of isolates^*a*^

					NBCI (*n* = 133)				All groups (*n* = 306)	
MLST-SCC*mec*	CC	*spa* type	% BCI (*n* = 36)	% SCI (*n* = 137)	% all NBCI	% STI (*n* = 78)	% RTI (*n* = 35)	% UTI (*n* = 20)	%	*n*
ST1-IVa	1	t127	0.0	8.0	4.5	6.4	0.0	5	5.6	17
ST7825-IVa	1	t127	0.0	0.7	0.0	0.0	0.0	0	0.3	1
ST5-IIa	5	t002, t045, t447, t548	27.8	31.4	24.1	9.0	51.4	35	27.8	85
ST5-IV	5	t2396	0.0	0.0	0.8	1.3	0.0	0	0.3	1
ST5- IVa	5	t010	2.8	3.6	2.3	1.3	2.9	5	2.9	9
ST5-IVc	5	t002	0.0	0.0	6.8	11.5	0.0	0	2.9	9
ST225-IIa	5	t003, t014, t045, t264, t564, t8447,14167, t21065	30.6	29.2	22.6	28.2	14.3	15	26.5	81
ST225-NT	5	t003	0.0	0.7	0.0	0.0	0.0	0	0.3	1
ST228-Ia	5	NT	0.0	0.0	0.8	1.3	0.0	0	0.3	1
ST1637-V	5	t13748	0.0	0.0	1.5	1.3	2.9	0	0.7	2
ST1728-IVa	5	t010	0.0	0.0	0.8	1.3	0.0	0	0.3	1
ST2626-V	5	t002	0.0	0.0	0.8	1.3	0.0	0	0.3	1
ST2883-IIa	5	t504, t1240, t4336	0.0	5.1	3.0	3.8	0.0	5	3.6	11
ST2883-NT	5	NT	0.0	0.7	0.0	0.0	0.0	0	0.3	1
ST7821-IIa	5	t045	0.0	0.7	0.0	0.0	0.0	0	0.3	1
ST7822-IIa	5	t002	0.0	0.0	1.5	2.6	0.0	0	0.7	2
ST7823-IVb	5	t008	0.0	0.0	0.8	1.3	0.0	0	0.3	1
ST7824-IIa	5	t003	0.0	0.7	0.0	0.0	0.0	0	0.3	1
ST7826-IIa	5	t003	0.0	0.0	0.8	0.0	2.9	0	0.3	1
ST7827-IIa	5	t003	5.6	0.0	0.0	0.0	0.0	0	0.7	2
ST7828-IIa	5	t003	2.8	0.7	0.0	0.0	0.0	0	0.7	2
ST8-IVa	8	t008	0.0	0.0	0.8	1.3	0.0	0	0.3	1
ST8-IVc	8	t6172	0.0	0.0	0.8	1.3	0.0	0	0.3	1
ST72-IVa	8	t148	0.0	0.0	0.8	1.3	0.0	0	0.3	1
ST254-IV	8	t009	0.0	0.0	0.8	1.3	0.0	0	0.3	1
ST22-IVa	22	t005, t022, t2933	13.9	5.8	4.5	2.6	5.7	10	6.2	19
ST22-IVh	22	t022, t790	2.8	2.2	2.3	0.0	2.9	10	2.3	7
ST30-IVa	30	t021, t2509	0.0	0.0	1.5	1.3	2.9	0	0.7	2
ST30-IVc	30	t685	0.0	0.7	0.0	0.0	0.0	0	0.3	1
ST30-V	30	t019	0.0	0.0	0.8	1.3	0.0	0	0.3	1
ST45-IVa	45	t390, t728, t808	2.8	0.0	2.3	2.6	2.9	0	1.3	4
ST45-IVb	45	t362	0.0	0.7	0.0	0.0	0.0	0	0.3	1
ST3154-IVa	45	t550	0.0	0.7	0.0	0.0	0.0	0	0.3	1
ST97-IVa	97	t2270, t11543	0.0	0.7	0.8	0.0	0.0	5	0.7	2
ST97-IVc	97	t359, t3380	5.6	4.4	3.0	3.8	2.9	0	3.9	12
ST599-XI	121	t5930	0.0	0.0	0.8	1.3	0.0	0	0.3	1
ST398-Vc	398	t011, t034, t571	2.8	2.2	3.8	3.8	5.7	0	2.9	9
ST88-IVa	none	t448, t690	0.0	0.7	3.8	2.6	2.9	10	2.0	6
ST152-V	none	t454	0.0	0.0	0.8	1.3	0.0	0	0.3	1
ST130-XI	none	t1048, t3256	2.8	0.0	1.5	2.6	0.0	0	1.0	3
ST7829-XI	none	t5090	0.0	0.0	0.8	1.3	0.0	0	0.3	1

^*a*^ MLST, multi locus sequence type; SCC*mec*, staphylococcal cassette chromosome *mec*; CC, clonal complex; BCI, blood culture isolates; SCI, surveillance cultures isolates; STI, isolates from soft tissues; RTI, isolates from respiratory tract; UTI, isolates from urinary tract; NBCI, non-blood clinical isolates; NT, non-typable; none, the clonal complexes of these strains are not known.

**Fig 1:**
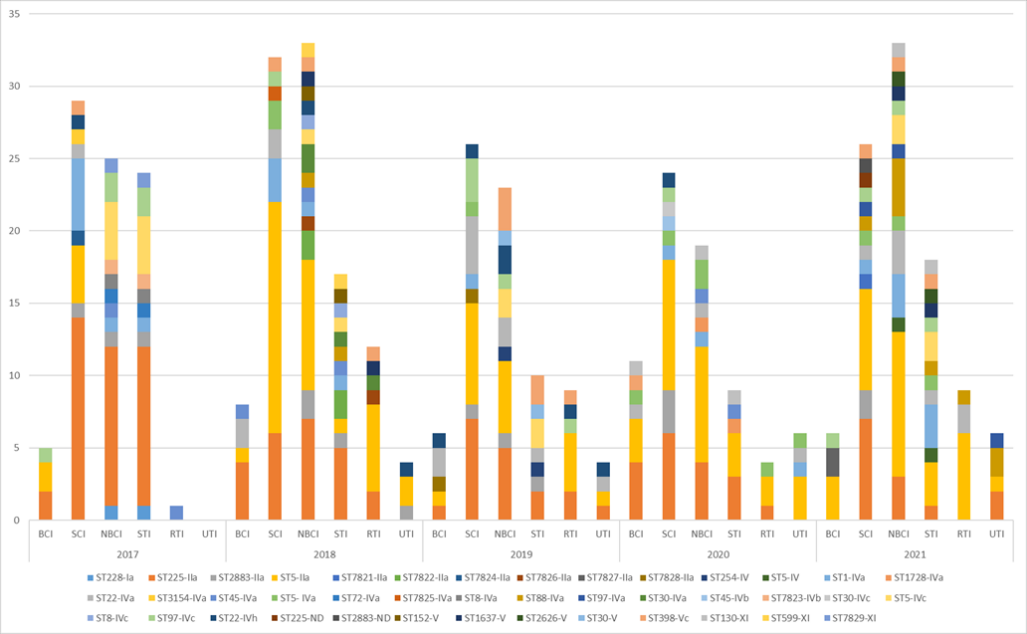
Distribution of MRSA isolates to genetic lineages across different sample origin and across a 5-year period (2017 to 2021). BCI, blood culture isolates; SCI, surveillance cultures isolates; NBCI, non-blood clinical isolates; STI, isolates from soft tissues; RTI, isolates from respiratory tract; UTI, isolates from urinary tract.

The two most prevalent genetic lineages exchanged their leading positions; ST225-IIa was the most prevalent lineage in 2017, and ST5-IIa was the leading lineage in 2021 (Fig. 1). The prevalence of CC5 lineages between 2017 and 2021 did not differ significantly (*P* = 0.279), but the increase in the prevalence of ST5-IIa and the decrease in the prevalence of ST225-IIa were significant (*P* = 0.0074 and *P* = 0.0002, respectively).

*Spa* typing revealed 49 *spa* types, among which t002 and t003, with 93 and 71 isolates (30.8% and 23.5%, respectively), were the most prevalent (Table 2). Out of 93 t002 isolates, 81 (87.1%) belonged to the ST5-IIa genetic lineage, and out of 71 t003 isolates, 64 (90.1%) belonged to the ST225-IIa genetic lineage. Twenty-six (55.3%) types had only one isolate assigned to a type, and 35 (74.5%) types were represented by less than 5% of all the isolates (Table S2). *Spa* typing increased the observed variability, especially within the two predominant lineages ST5-IIa and ST225-IIa (Table 2).

### Clustering of HA-MRSA, CA-MRSA, and LA-MRSA in hospitals and hospital-associated facilities

A total of 212 (69.7%) isolates were from hospitalized patients and 94 (30.3%) from non-hospitalized patients. The non-hospital samples originated from emergency medical services (EMS) (38 patients, 12.5%), hospital outpatient clinics (HOC) (22 patients, 7.2%), long-term healthcare institutions (LTHCI) (13 patients, 4.3%), primary healthcare centers (PHCC) (12 patients, 3.9%), dialysis centers (DC) (4, 1.3%), and spas providing medical services (SP) (3 patients, 1.0%). Samples containing HA-MRSA, CA-MRSA, and LA-MRSA were collected during hospitalization in 72.7%, 66.7% and 63.0% of cases, respectively (Table S3). The largest proportion of HA-MRSA isolates (49.7%) was detected in surveillance cultures, while the largest proportion of CA-MRSA (54.0%) and LA-MRSA isolates (53.7%) was recovered from non-blood clinical samples. The majority of isolates from clinical samples originated from soft tissue (Table 3).

**TABLE 3:** The proportions of HA-MRSA, CA-MRSA, and LA-MRSA within different groups of isolates^*a*^

Group of isolates		HA-MRSA		CA-MRSA		LA-MRSA	
	*n*	*n*	%	*n*	%	*n*	%
BCI	36	24	12.8	8	12.7	4	7.4
STI	78	35	18.7	19	30.2	24	44.4
RTI	35	24	12.8	8	12.7	3	5.6
UTI	20	11	5.9	7	11.1	2	3.7
NBCI	133	70	37.4	34	54.0	29	53.7
SCI^*b*^	135	93	49.7	21	33.3	21	38.9
BCI and NBCI	169	94	50.3	42	66.7	33	61.1
BCI and STI	114	59	31.6	27	42.9	28	51.9

^*a*^ BCI. blood culture isolates; NBCI. non-blood clinical isolates; SCI. isolates from surveillance cultures; STI. isolates from soft tissues; RTI; isolates from respiratory tract; UTI. isolates from urinary tract; HA-MRSA. hospital-acquired MRSA; CA-MRSA. community-acquired MRSA; LA-MRSA. livestock-associated MRSA.

^*b*^ 2 out of 137 isolates could not be categorized as SCCmec could not be determined.

**Fig 2:**
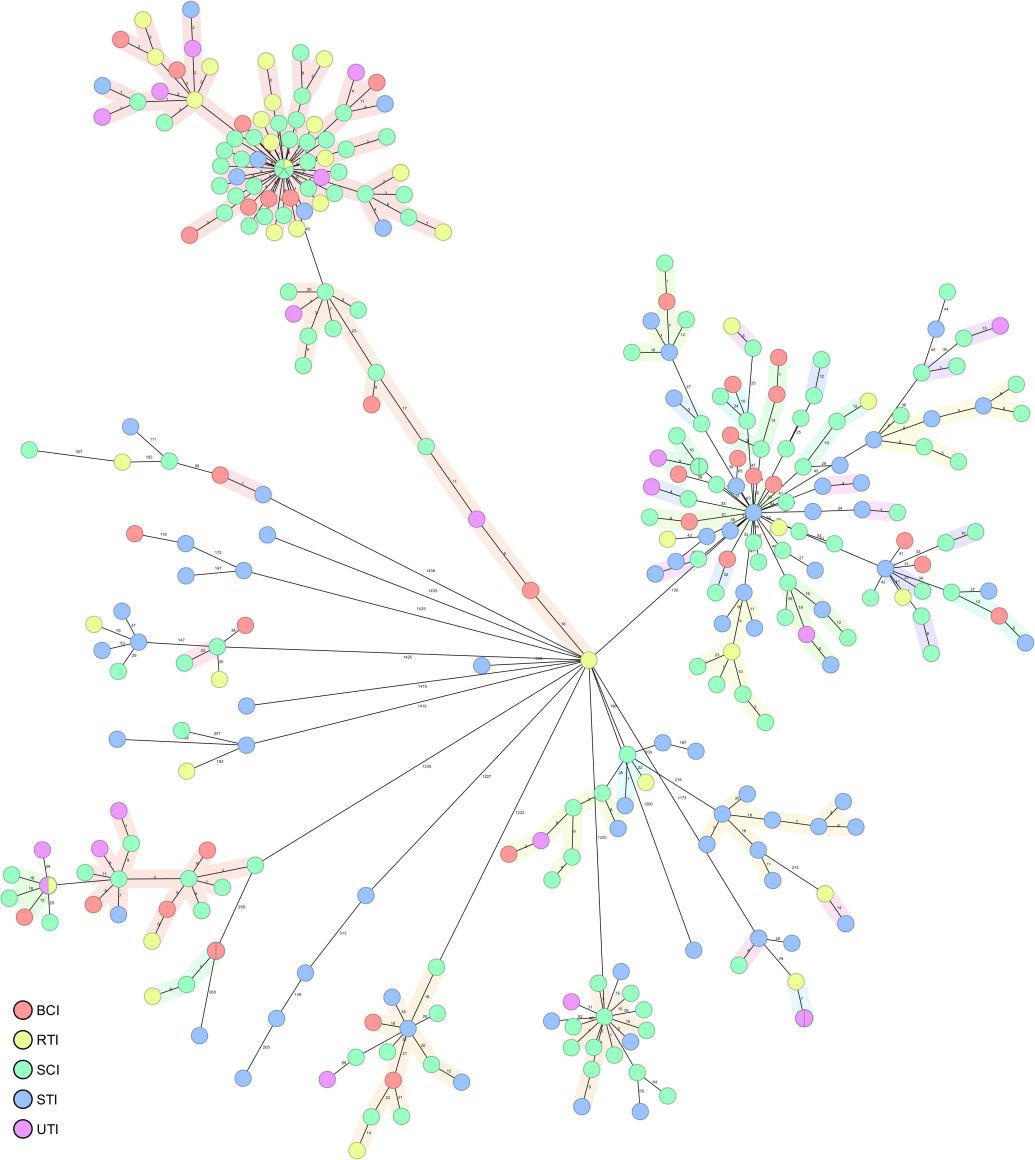
Minimum spanning tree of 306 MRSA included in the study. MST clonal clusters (with maximum allelic distance of 24 alleles) are surrounded by color. Each circle represents one or more isolates with a unique cgMLST profile. Numbers connecting circles denote the number of allelic differences. Circles are colored according to isolate group (BCI, blood culture isolates; SCI, isolates from surveillance cultures; STI, isolates from soft tissues; RTI; isolates from respiratory tract; UTI, isolates from urinary tract).

The analysis of allelic differences using core genome multi-locus sequence typing (cgMLST) resulted in the generation of 38 clusters, with 2–72 isolates in each, adhering to a cluster distance threshold of 24 (Fig. 2). Seventy-four isolates (24.2%) were identified as singletons. The temporal distribution of clusters varied, with 6 clusters lasting for 1 year, 11 spanning over 2 years, 6 over 3 years, 7 over 4 years, and 8 having representatives scattered over a 5-year period.

**Fig 3:**
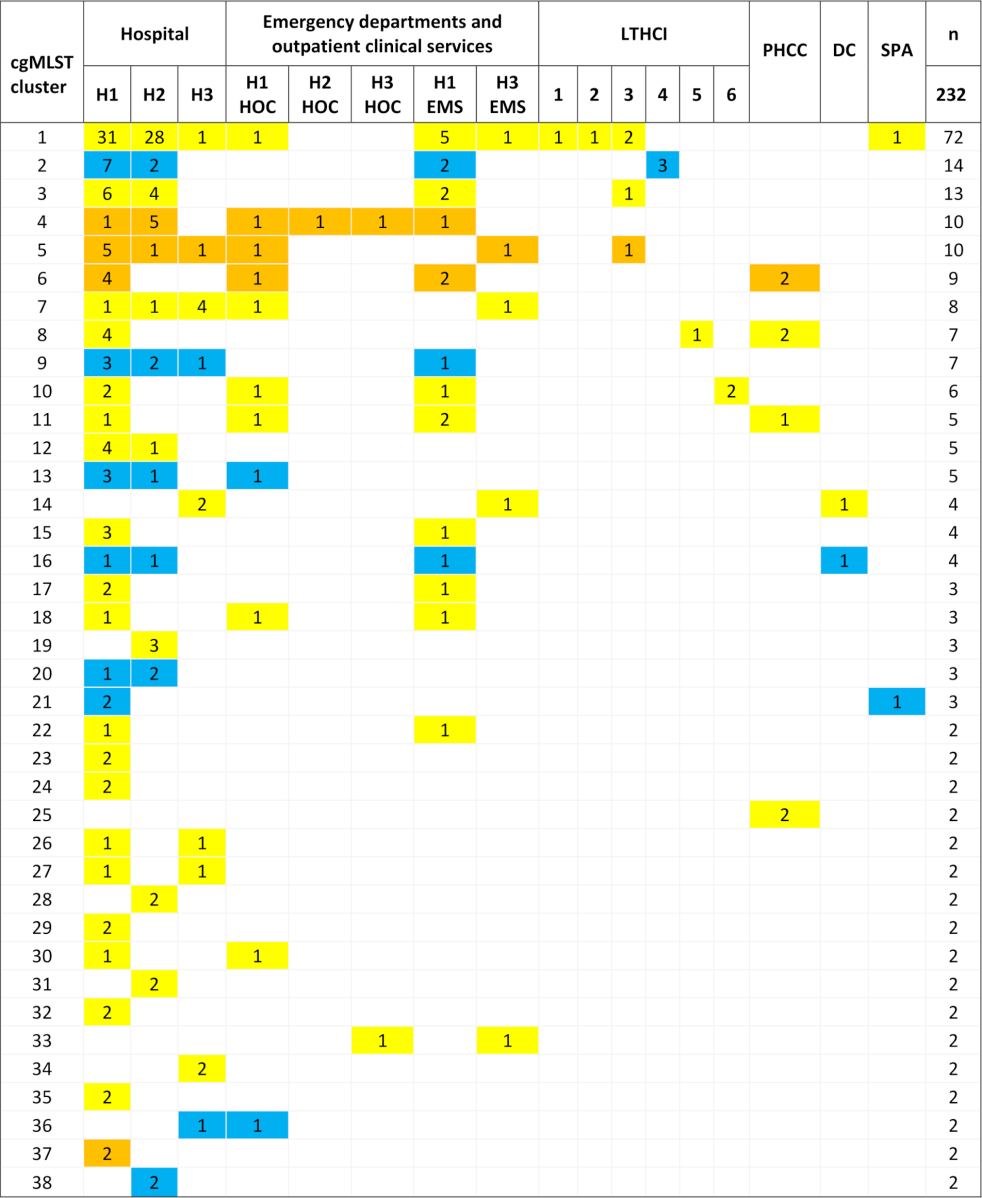
cgMLST clusters of MRSA isolates across different healthcare environments. H1, H2, H3, hospitals; EMS1, 3, emergency medical services; HOC1, 2, 3, hospital outpatient clinics; LTHCI1, 2, 3, 4, 5, 6, long-term healthcare institutions; PHCC, primary healthcare centers; DC, dialysis centers; SP spas with medical services. Yellow color represents HA-MRSA clusters, orange LA-MRSA clusters, and blue CA-MRSA clusters. The numerical values within the fields represent the count of isolates from the respective sampling site within each cluster.

LA-MRSA isolates formed 4 clusters, CA-MRSA isolates 8 clusters, and HA-MRSA isolates 26 clusters. In all but one case, the majority of isolates originated from the hospital environment (Fig. 3). Isolates from surveillance samples had representatives in 33 clusters, blood culture isolates in 14 clusters, and non-blood clinical isolates in 30 clusters (Table S4).

### Virulence genes in the entire collection and distribution within the genetic lineages

Ninety-six virulence genes were identified, and their prevalence ranged from 0.7% to 100% across the isolates. Thirty-four (35.4%) genes were present in all isolates, and 58 (60.4%) were present in more than 99% of the isolates (Table S5). The lowest proportion (89.6%) of all virulence genes observed in the study was identified in the group of blood culture isolates. The complete set of virulence genes was obtained from the group of soft tissue infection isolates and consequently from the entire group of non-blood clinical isolates (Table 4).

**TABLE 4:** Contribution of different groups of isolates to detection of genetic traits of MRSA^*a*^

Group of isolates	Isolates	SCC*mec* types		MLST		MLST-SCC*mec* profiles		*spa* types		Resistance phenotypes		Virulence genes		Resistance genes	
	*n*	*n*	%	*n*	%	*n*	%	*n*	%	*n*	%	*n*	%	*n*	%
BCI	36	6	60.0	9	30.0	11	28.2	10	20.4	7	24.1	86	89.6	35	61.4
STI	78	9	90.0	23	76.7	28	71.8	32	65.3	17	58.6	96	100.0	47	82.5
RTI	35	6	60.0	10	33.3	12	30.8	14	28.6	9	31.0	92	95.8	37	64.9
UTI	20	3	30.0	7	23.3	9	23.1	7	14.3	5	17.2	92	95.8	26	45.6
NBCI	133	10	100.0	24	80.0	31	79.5	39	79.6	22	75.9	96	100.0	52	91.2
SCI	137	6	60.0	15	50.0	20	51.3	25	51.0	15	51.7	93	96.9	46	80.7
BCI and NBCI	169	10	100.0	26	86.7	33	84.6	32	65.3	26	89.7	96	100.0	53	93.0
BCI and STI	114	10	100.0	25	83.3	31	79.5	39	79.6	21	72.4	96	100.0	51	89.5
BCI and SCI	173	8	80.0	17	56.7	23	59.0	27	55.1	17	58.6	94	97.9	48	84.2
All	306	10		30		39		49		29		96		57	

^*a*^ BCI, blood culture isolates; SCI, isolates from surveillance cultures; STI, isolates from soft tissues; RTI, isolates from respiratory tract; UTI, isolates from urinary tract; NBCI, non-blood clinical isolates; SCC*mec*, staphylococcal cassette chromosome *mec*; MLST, multi locus sequence type.

The predominant lineages ST225-IIa and ST5-IIa differed significantly in the frequencies of five virulence genes, of which one (*fnbB*) was in favor of ST225-IIa and four (*chp*, *sak*, *coa*, and *sdrE*) in favor of ST5-IIa (Table S6).

Genes present in less than 10% of isolates were restricted to less frequently represented genetic lineages. No HA-MRSA isolates were positive for PVL genes, *tsst1 *and *vWbp* and the majority of enterotoxin genes (*seb*, *sec*, *seh*, *selk*, *sell*, and *selq*). Only one HA-MRSA isolate harbored *sea* genes.

Twenty isolates were positive for the Panton-Valentine leucocidin gene. They formed three complete cgMLST clusters, the first consisting of nine LA-MRSA isolates with the ST5-IVc-t002 profile (cluster 6) and clusters 21 and 36 consisting of three and two CA-MRSA isolates, respectively, all with the ST88-IVa-t690 profile. Six PVL-positive isolates were singletons and were the sole representatives of ST-SCC*mec-spa* profiles ST8-IVa-t008, ST8-IVc-t6172, ST22-IVa-t005, ST88-IVa-t448, ST152-V-t454, and ST7823-IVb-t008.

Of the nine *tsst-1*-positive isolates, four belonged to one cluster (cluster 16) composed of the ST22-IVa-t2933 lineage and five were singletons, each being the sole representative of five ST-SCC*mec-spa* profiles ST-45-IVa-t728, ST30-IVa-t2509, ST599-XI-t9925, ST30-IVc-685, and ST22-IVa-t005.

Five *mecC-positive* isolates, ST130-XI-t1048 (two isolates), ST130-XI-t3256, ST599-XI-t5930, and ST7829-XI-t5090, were identified. They all lacked the *sak* and *scn* genes, and only one had the *chp* gene. The gene for exfoliative toxin (*etb*) was only found in two isolates with the ST130-XI-t1048 profile. ST599-XI-t5930 had a unique virulence profile that was positive for *sac*, *sell*, *set16*, *set26*, and *sdrD* and negative for *esaE. esaD*, *esxB*, *esxc*, *esxD*, *cap8H*, *cap8I*, *cap8J*, and *cap8K*, compared with other *mecC*-positive isolates.

In 2017, out of 5,568 possible virulence genes hits, 4,459 (80.1%) were present, and in 2021, out of 6,240 hits, 4,943 (79.2%) were present. Therefore, the total virulence gene load did not differ significantly between the beginning and the end of the observation period (*P* = 0.2425).

### Resistance genes and concordance with resistance phenotypes

Fifty-seven resistance genes conferring 22 different resistance mechanisms were present in our isolate pool (Table S4). Each individual gene was carried by 0.3% to 98.4% of the 306 isolates (Table S7). Blood culture isolates carried 61.4% of different resistance genes, isolates from urinary tract infections 45.6%, isolates from respiratory tract infections 64.9%, isolates from soft tissue infections 82.5%, and surveillance culture isolates 91.2% of the resistance genes (Table 4).

In 2017, 636 resistance genes out of 3,249 possible hits (19.6%) were detected, and in 2021, 750 (20.2%) out of 3,705 hits were detected. The total burden of resistance genes therefore did not differ significantly between the start and end of the observation period *P* = 0.4835).

As expected, all isolates were resistant to penicillin and methicillin, and 43 (14.1%) isolates were resistant only to these two antibiotics. They all carried the genetic traits of LA or CA-MRSA. Resistance to ciprofloxacin, erythromycin, and clindamycin exceeded 70%, and inducible clindamycin resistance (iMLS_b_) was detected in 39.7% of isolates. Resistance to tetracycline and gentamycin was detected in 10.4% and 5.5% isolates, respectively. Only one isolate was resistant to trimethoprim–sulfamethoxazole and two to mupirocin. No vancomycin resistance was detected.

Twenty-nine phenotypic resistance profiles were identified, 4 of which were represented by more than 10 isolates, type 2 by 91 (29.7%) isolates, type 3 by 11 (3.6%) isolates, type 5 by 43 (14.1%) isolates, and type 6 by 105 (34.3%) isolates. The most prevalent types 2 and 6 differ only in their iMLS_b_ profile. Twenty resistotypes were represented by less than one isolate per year and 15 by only one isolate in the 5-year period (Table 5).

**TABLE 5:** Prevalence of resistance and resistance profiles among HA-MRSA, CA-MRSA, and LA-MRSA isolates^*a*^

Resistance profile	Susceptibility											All isolates (*n* = 306)		HA-MRSA (*n* = 187)		CA-MRSA (*n* = 63)		LA-MRSA (*n* = 54)	
	P	OX	CIP	GM	E	CC	iMLSb	TE	SXT	VA	MUP	*n*	%	*n*	%	*n*	%	*n*	%
1	R	R			R	R		R				3	1.0					3	5.6
2	R	R	R		R	R						91	29.7	87	46.5				
3	R	R						R				11	3.6			4	6.3	7	13.0
4	R	R	R	R	R	R						5	1.6	5	2.7				
5	R	R										43	14.1			17	27.0	26	48.1
6	R	R	R		R	R	POS					105	34.3	87	46.5	19	30.2	1	1.9
7	R	R			R	R						2	0.7	1	0.5	1	1.6		
8	R	R	R		R	R		R				1	0.3	1	0.5				
9	R	R			R							1	0.3			1	1.6		
10	R	R	R	R								3	1.0	1	0.5			2	3.7
11	R	R			R			R				1	0.3			1	1.6		
12	R	R			R	R	POS					6	2.0			3	4.8	3	5.6
13	R	R	R									8	2.6	1	0.5	7	11.1		
14	R	R		R								4	1.3			4	6.3		
15	R	R	R	R	R	R		I				1	0.3	1	0.5				
16	R	R		R				R				1	0.3			1	1.6		
17	R	R				R		R				2	0.7					2	3.7
18	R	R	R		R	R		I				1	0.3	1	0.5				
19	R	R			R	R	POS	R				6	2.0			1	1.6	5	9.3
20	R	R	R	R				R				1	0.3					1	1.9
21	R	R	R		R							2	0.7	2	1.1				
22	R	R							R			1	0.3			1	1.6		
23	R	R				I		R				1	0.3					1	1.9
24	R	R		R	R	R		R				1	0.3					1	1.9
25	R	R	R		R	R	POS				R	1	0.3			1	1.6		
26	R	R	I		R	R	POS	R				1	0.3					1	1.9
27	R	R									I	1	0.3					1	1.9
28	R	R	R		R	R	POS	R				1	0.3			1	1.6		
29	R	R		R	R	R	POS					1	0.3			1	1.6		
*n* of R/I/POS	306	306	221	17	230	229	121	32	1	0	2	na	na	na	na	na	na	na	na
% of R/I/POS	100	100	72.2	5.6	75.2	74.8	39.5	10.5	0.3	0.0	0.7	na	na	na	na	na	na	na	na

^*a*^ R, resistant; I, intermediate; POS, positive; P, penicillin; OX, oxacillin; CIP, ciprofloxacin; GM, gentamycin; E, erythromycin; CC, clindamycin; iMLSb, inducible macrolide-lincosamide-streptogramin B resistance; TE, tetracycline; SXT, trimethoprim-sulfamethoxazole; VA, vancomycin; MUP, mupirocin; HA-MRSA, hospital-acquired MRSA; CA-MRSA, community-acquired MRSA; LA-MRSA, livestock-associated MRSA; na, not applicable.

For 10 antibiotics tested phenotypically, the agreement between phenotype and genotype was 100% for methicillin, vancomycin, and mupirocin; 99.2% for gentamycin; 97.7% for ciprofloxacin; and 86.9% for trimethoprim–sulfamethoxazole. The agreement between the methods for tetracycline was 98.4% when *tetK* and *tetM* genes were taken into consideration and only 11.1% when the *tet38* gene was also included. The b*laZ* gene was detected in 204 (65.8%) of the isolates.

### The diversity of MRSA genetic lineages among different groups of samples

To evaluate the informative value of the different groups of samples/isolates and their combinations, the proportions of the detected characteristics were compared and *P* values were calculated (Tables 4 and 6). Isolates from blood cultures demonstrated lower informativeness compared with surveillance culture isolates, all clinical isolates combined, and the group of soft tissue isolates. However, they were more informative than isolates from respiratory and urinary tract samples. Blood culture isolates were able to detect 21.3% of *spa* types, 24.1% of resistance phenotypes, 28.2% of MLST-SCC*mec* profiles, 30.0% of MLST types, 55.6% of SCC*mec* types, 89.6% of virulence genes, and 61.4% of resistance genes.

**TABLE 6:** Significance of differences in ability of different sample groups to detect genetic traits of MRSA isolates^*a*^

Group of isolates	SCC*mec* types	MLST	MLST-SCC*mec* profiles	*spa* types	Resistance phenotypes	Virulence genes	Resistance genes
	*P* -values						
pBCI < pSTI	0.061	0.000	0.000	0.001	0.004	0.001	0.006
pBCI < pRTI	0.500	0.391	0.402	0.174	0.278	0.048	0.349
pBCI < pUTI	0.911	0.720	0.698	0.788	0.742	0.480	0.954
pBCI < pNBCI	0.013	0.000	0.000	0.000	0.000	0.001	0.000
pBCI < pSCI	0.500	0.057	0.019	0.001	0.015	0.022	0.012
pBCI < pBCI + NBCI	0.013	0.000	0.000	0.000	0.000	0.001	0.000
pBCI < pBCI + STI	0.013	0.000	0.000	0.000	0.000	0.001	0.000
pBCI < pBCI + SCI	0.165	0.019	0.003	0.000	0.004	0.009	0.003
pSCI < pSTI	0.061	0.016	0.031	0.076	0.299	0.040	0.405
pSCI < pRTI	0.500	0.905	0.967	0.988	0.945	0.650	0.971
pSCI < pUTI	0.911	0.984	0.995	1.000	0.997	0.650	1.000
pSCI < pNBCI	0.013	0.007	0.004	0.001	0.028	0.040	0.053
pBCI + STI < pBCI + NBCI	NA	0.359	0.278	0.943	0.047	NA	0.254
pBCI + SCI < pBCI + STI	0.068	0.012	0.025	0.005	0.135	0.078	0.203
pBCI + SCI < BCI + NBCI	0.068	0.005	0.006	0.151	0.003	0.078	0.127

^*a*^ BCI, blood culture isolates; SCI, isolates from surveillance cultures; STI, isolates from soft tissues; RTI, isolates from respiratory tract; UTI, isolates from urinary tract; NBCI, non-blood clinical isolates; SCC*mec*, staphylococcal cassette chromosome *mec*; MLST, multi locus sequence type.

Clinical samples performed better than isolates from surveillance cultures, and the same applies to their combination with isolates from blood cultures. Alone, clinical samples were able to detect 79.5% of MLST-SCC*mec* profiles, 75.9% of resistance phenotypes, 80.0% of MLST types, 83.0% of *spa* types, 91.2% of resistance genes, 100.0% of SCC*mec* types, and 100% of virulence genes. Adding all the clinical samples to blood culture isolates instead of only adding isolates from soft tissues was not a significantly better approach (Table 6). The addition of soft tissue isolates to blood cultures instead of surveillance cultures proved to be a better strategy for investigating the clonal structure of the MRSA population but not for detecting the burden of virulence and resistance genes.

## DISCUSSION

Here, we present the first MRSA WGS data for Slovenia derived from a comprehensive strain collection from a larger Slovenian region and spanning across 5 years. The collection was subsequently used to define the sample groups that would best represent genomic diversity and could be suitable for an improved and updated MRSA surveillance protocol.

### Clonal structure of MRSA in the region

Of the 9 established European HA-MRSA clones, 12 CA-MRSA clones, and 6 LA-MRSA clones, we detected 3 (ST5-II, ST225-II, and ST228-I), 6 (ST5-IV, ST8-IV, ST22-IV, ST30-IV, ST88-IV, and ST45-IV), and 4 (ST398-V, ST97-IV, ST1-IV, and ST130-XI), respectively (20). The presence of 39 MLST ST-SCC*mec* and 60 MLST ST-SCC*mec-spa* profiles along with a high proportion of singletons in cgMLST clustering (24.2%) within a relatively limited geographic area is likely the consequences of previously described factors such as independent introduction of clones related to travel and migration, local diversification of existing MRSA lineages, horizontal gene transfer to locally successful MSSA clones, environmental factors including antibiotic consumption rates, and infection control practices (20, 21). Up to 65% of notified MRSA cases was reported to be migration and travel related, and up to 85% cases can be community acquired (21, 22). Despite drastic differences in local MRSA epidemiology, epidemic waves of successful clones following one another are common characteristics of different environments. Modeling has demonstrated that they can be interrupted by early interventions supporting the concept of prompt detection of emerging clones (23).

High predominance of isolates belonging to CC5 and local diversification within the clone was observed, with the ST5-IIa lineage replacing ST255-IIa in the leading position during the 5-year observation period. Both belong to the Rhine-Hesse MRSA clone, which has been present in Europe since 1995 (24). ST5 has been among the most prevalent clones causing hospital-acquired infections worldwide, and it is the presumed ancestor of CC5 (20, 25, 26). ST225-IIa probably diverged from ST5 in the early 1980s, and it is believed that it was introduced to Europe from the United States through a single transmission event (27). In Germany, it was detected in the Euregio Meuse-Rhine Region, in 2003, and spread rapidly during the next 10 years particularly in central European countries (11, 28, 29). In the first survey of invasive infections, which was conducted in 26 European countries from September 2006 to February 2007, all isolates from Slovenia belonged to *spa* type 041. ST225-t003 and ST5-t002 were already the fourth and fifth most prevalent lineages in Europe at that time and remained among the top five most common MRSA *spa* types also in the second survey in 2011 (10, 11). The first description of ST225-II-t003 and ST2883-IIa-t4336 from Slovenia dates back to 2010 (28, 30), but the first description of ST5-IIa-t002 could not be found with certainty as either the SCC*mec* type was not determined or the isolates belonged to ST5-IV-t002 (31, 32).

ST225-IIa and ST5-IIa isolates were all resistant to erythromycin and ciprofloxacin, which has been identified as a marker for the success of different MRSA clones in France, the Netherlands, and the United Kingdom. MRSA incidence was found to correlate with the total consumption of these antibiotics in the community (17). In Slovenia, consumption, measured in DDDs per 1,000 inhabitants per day, is right between France and the Netherlands (33) and both parameters, MRSA incidence from blood cultures and consumption of quinolones and macrolides in community, have been stable for the last 10 years (18, 33). Therefore, the unchanged selective pressure could have been the main reason for the persistence and dominance of the same clonal complex and the differences in the ability to evade the immune system and to adhere might explain the fact that ST5 has taken the leading position but has not completely displaced ST225. In Germany, it has previously been described that these two lineages were able to coexist for decades (24). The consumption of quinolones in the community may also have contributed to the selection of other successful lineages in this region, as four of the five top ranking clones among all isolates (ST2-IIa, ST225-IIa, ST22-IVa, and ST1-IVa) and three of the top five among blood culture isolates (ST2-IIa, ST225-IIa, and ST22-IVa) were resistant to ciprofloxacin.

In our study, ST22 isolates proved to be a highly diverse population harboring two different staphylococcal cassettes (IVa, IVh), displaying six phenotypic resistance profiles, belonging to four *spa* types (t005, t022, t790, and t2933), and forming three cgMLST clusters. Based on the genetic profiles of our isolates, we can conclude that they likely belong to all three global clades—A represented by the EMRSA-15 clone which consists only of SCC*mec* IVh-positive–*tsst1*-negative strains; B represented by the Gaza clone, composed mainly of SCC*mec* IVa–*tsst1*-positive strains; and C, which is composed mainly of PVL-positive strains (34). The first ST22 isolates belonging to t011, t020, and t1218 were detected in Slovenia in 2010 and isolates belonging to ST22-t223 and ST22-t022 in 2015 (31, 32).

### Less frequently detected clones

Representatives of CC1-ST1-IVa and one isolate belonging to a novel ST7825-IVa were all *spa* typed as t127 and PVL negative. They can be considered as livestock adapted as their human counterparts are PVL positive (20). In addition, they were all positive for *seh* and *vWbp* genes. Being livestock adapted probably explains the observation that the lineage did not cause any blood stream infections, although it ranked fourth among all isolates and was present throughout the observation period. Greater diversity was observed in antibiotic resistance with nine different resistance profiles detected and in cgMLST clustering where eight isolates were singletons, which could mean that this clone is present in different animal herds or farms in Slovenia or has been introduced many times from other countries. It was reported in cattle in the Czech Republic and in pigs in Italy, Finland, and Spain (20, 35, 36). Among bloodstream infections, ST1-IVa ranked 20th and 11th in the two European surveys. In Slovenia, it has been described already in 2010 (30).

In our study, the prevalence of *mec*A was 98.4%. Among these, 91.3% belonged to three SCC*mec* types (IIa, Iva, and IVc), while the remaining six SCC*mec* types were represented by less than 3% of isolates each (Table 1). The prevalence of *mec*C-positive isolates, accounting for only 1.6%, is comparable to the low prevalence reported in Slovenia and globally (20, 31, 32, 37). In Slovenia, a significant increase in the prevalence of *mecC*-positive isolates from 1.52% to 8.2% was observed between 2006 and 2015, but the data are not comparable since the isolates from the previous studies had been selected based on their resistance profile (31, 32). All five *mecC*-positive isolates from our study (four from SSI; one from BI) were singletons with distinctive virulence profiles and isolated oxacillin resistance, which was found to be characteristic especially for ST130 isolates (38). All lacked the *sak* and *scn* genes, and only one had the *chp* gene, possibly indicating an animal origin. Septic events with ST130 strains have been described previously but are not frequent (37, 39, 40). The ST130-related *spa* types observed in our study—t1048, t3256, t5090, and t5930—are rarely described, as only 10 entries have been made in the Ridom spaserver database (http://spaserver.ridom.de/, last accessed 10 November 2023) since 2011 mainly from Germany, the Czech Republic, and Denmark.

The two other globally established LA-MRSA clones CC97 with 4.6% and CC398 with 2.9% ranked fifth and seventh, respectively, in our strain collection. Among the blood culture isolates, those with the ST97-IVc profile ranked fourth to fifth. ST398-Vc was represented by one isolate as well as five other genetic lineages (ST130-XI, ST22-IVh, ST45IVa, ST5-IVa, and ST7828-IIa). The prevalence of CC398 among presumptive CA-MRSA isolates from Slovenia was reported to be significantly higher between 2010 and 2015, and the increase from 15.2% to 27.5% was found to be statistically significant (32). With the exception of t571, t034 and t011 have been already present since 2010. CC97 isolates were detected in Slovenia in 2014 and represented 3.8% of presumptive CA-MRSA isolates in 2015 (32). *Spa* type t359 was the most prevalent in the current and previous Slovenian study. All CC398 and CC97 isolates from our study were negative for *tsst1*, PVL, and enterotoxin genes. CC97 showed stronger adaptation to the human host, as all but one isolate were *sak-* and *scn*-positive than the isolates of CC398, which were all negative for these two IEC genes. Since 78.3% of these isolates were from hospitalized patients and formed two cgMLST clusters in the hospital environment and 56.5% were from clinical isolates, we can conclude that LA-MRSA clones are well established in the hospital environment and frequently cause infections. Other studies confirmed the migration of LA-MRSA to hospitals (41, 42). At the same time, we can conclude that in Slovenia, due to the lack of a surveillance system, the time of emergence of these isolates in the human population and the point of entry into the hospital environment was largely overlooked.

### Possible upgrading of sampling strategy

Our study confirms previous observations that an improved sampling strategy may be needed for MRSA surveillance. We found that isolates from blood cultures provide an incomplete picture of the total MRSA population in studying epidemiology or assessing virulence and resistance gene burden. The information obtained from blood culture isolates was significantly lower, with greater than 95% confidence for all parameters examined. While blood culture isolates provided insights into the most frequently detected clones, majority of clones and clusters as well as some important virulence and resistance genes were missed.

Based on our findings, we recommend including other types of samples in surveillance systems for the early detection of emerging clones, understanding their entry points into populations, and studying the clonal structure of the MRSA population. In particular, isolates from soft tissues proved to be the most informative candidates.

Despite the limited geographical area covered in our study, the demonstrated genetic diversity of the isolates suggests that this factor did not significantly influence the results. However, the small overall number of isolates, blood culture isolates, urinary tract isolates, and respiratory isolates could be limiting factors. With a bigger overall sample, we would probably detect additional genetic lineages, and with bigger subgroups, the proportions of detected traits and the informative value of each subgroup in relation to others could be different. Further studies are needed to determine whether under similar conditions, such as selective pressure due to antibiotic consumption, hygienic practices, and the specific structure of MRSA and its host population, an increase in sample size would maintain differences in the informative values of the sample groups.

Given the declining prevalence of MRSA infections in many European countries, including those participating in the EARSS (24 out of 30 European countries have already lowered MRSA prevalence among *S. aureus* blood culture isolates below 25%), the value of blood culture isolates is expected to decline, particularly in middle- and high-income countries (18). This highlights the need for continuous adaptation and optimization of MRSA surveillance strategies in response to evolving epidemiological trends.

In summary, our study in a selected Slovenian region revealed a diverse MRSA landscape with two predominant and dynamically changing genotypes. Notably, analysis of our data set suggests that bloodstream infections may not comprehensively reflect the entirety of MRSA diversity, underscoring the importance of considering alternative sources of MRSA surveillance and analysis. Among the groups studied, isolates from soft tissues were found to be the most representative of the overall genetic diversity of MRSA, as they comprise a substantial part of the resistance and virulence gene pools. Therefore, we suggest including soft tissue samples in surveillance protocols as they provide valuable insights into the broad genetic landscape of MRSA.

## MATERIALS AND METHODS

### Strain selection

A total of 306 isolates, selected from the MRSA collection at the Department of Microbiology Celje, Centre for Medical Microbiology, National Laboratory of Health, Environment, and Food spanning the years 2017 to 2021, were included in the study. The laboratory serves at least 17% of the Slovenian population living in 2 of 12 geographical and statistical regions. Samples are sent to the laboratory by three hospitals and other healthcare providers. The study was approved by institutional Ethic Committee of Medical Faculty, University of Maribor (038/2022/3-401).

The laboratory routinely stores the first isolate per patient per year and all isolates from blood cultures. The compliance with this rule has been 100% for blood culture isolates and 84.5% for other isolates. All the isolates (altogether 36) from blood cultures (BCI group) and 10% (every tenth) of other isolates were included. Isolates from clinically relevant samples (CI group) were subdivided in three groups with 78 isolates representing soft tissue infections (STI group), 35 isolates representing respiratory infections (RTI group), and 20 isolates representing urinary tract infections (UTI group). Additional 137 isolates were from surveillance cultures (SCI group). Duplicated isolates per patient were excluded by giving priority to isolates form blood cultures.

### Phenotypic characterization

The identification was confirmed after re-cultivation with the matrix-assisted laser desorption ionization–time of flight–MALTI-TOF microflex LRF (Bruker).

Susceptibility testing for penicillin, methicillin, gentamycin, ciprofloxacin, erythromycin, clindamycin, tetracycline, vancomycin, trimethoprim–sulfamethoxazole and mupirocin was performed with the disc diffusion method in accordance with The European Committee on Antimicrobial Susceptibility Testing (EUCAST) standards (43). Isolates were assigned consecutively to a new resistotype if they were resistant or intermediate resistant to at least one antibiotic differently as in all the previous isolates. Inducible or constitutive expression of clindamycin resistance was considered as a difference in resistance profile.

### Whole-genome sequencing and analysis

DNA was extracted from the overnight culture on the blood agar medium using the QIAamp DNA Mini Kit (Qiagen, Germany), following protocol for isolation of genomic DNA from Gram-positive bacteria. DNA concentration was quantified using the PicoGreen dsDNA Assay Kit (Waltham, US) or the Qubit Fluorometer (Invitrogen). Libraries were prepared using the NEBNext Ultra II FS DNA Library Prep Kit for Illumina (New England Biolabs, UK) and subjected to 2 × 150 bp paired-end sequencing on the NextSeq2000 platform (Illumina, US) following the manufacturer’s protocol. Genomic sequencing for 36 samples was performed by the commercial provider Novogene (Novogene Co. Ltd., UK) on the Illumina Novaseq 6000 platform according to their standard protocols. The sequenced reads were first trimmed with Trimmomatic version 0.39 (44) with the following settings: ILLUMINACLIP:NEBPE-PE.fa:2:30:10, LEADING:10, TRAILING:10, SLIDINGWINDOW:4:20, and MINLEN:40. Next, trimmed reads were *de novo* assembled into contigs using SPAdes version 3.13.1 (45) with the -careful option. All other options were left as default.

Antimicrobial resistance genes and point mutations were identified using the AMRFinderPlus version 3.10.45 (46), with database version 2022-10-11.2. The ID threshold was set on 80% or 90%, and the coverage threshold was set on 60% or 50%. Virulence factors were analyzed using the Abricate software version 1.0.1 (https://github.com/tseemann/abricate) (47) with the Virulence Factor Database version 2021-Mar-27, using an ID threshold of 85% (48).

Staphopia-sccmec software version 1.0.0 (49) (https://github.com/staphopia/staphopia-sccmec) was used to determine the SCC*mec* types in *mecA*-positive isolates and SCCmecFinder software version 1.2 (https://cge.food.dtu.dk/services/SCCmecFinder/), with default settings, to determine the SCC*mec* type in isolates carrying *mecC* gene.

Ridom SeqSphere+ 9.0.2 (50) software was used to determine STs based on the multi-locus sequence typing scheme and spa types (*in silico* or by Sanger sequencing of PCR amplified spa gene repeat region) (51) and to analyze genetic relatedness of isolates, based on a cgMLST.

### Statistical analysis

Genes associated with a specific lineage and prevalences between years were compared using Fisher’s exact test at a 5% significance threshold.

For comparison between the groups, a one-sided test of difference in proportions was employed to appropriately address the directionality and *P* values calculated.

## Data Availability

Sequences for all sequenced isolates are available in Sequence Read Archive under accession number PRJNA1059797.

## SUPPLEMENTAL MATERIAL

Supplemental tables spectrum.00140-24-s0001.xlsx Tables S1-S7.

ASM does not own the copyrights to Supplemental Material that may be linked to, or accessed through, an article. The authors have granted ASM a non-exclusive, world-wide license to publish the Supplemental Material files. Please contact the corresponding author directly for reuse.
